# Deletion of Coagulation Factor IX Compromises Bone Mass and Strength: Murine Model of Hemophilia B (Christmas Disease)

**DOI:** 10.1007/s00223-021-00872-x

**Published:** 2021-06-12

**Authors:** Emily A. Larson, Hillary J. Larson, Jason A. Taylor, Robert F. Klein

**Affiliations:** 1 Portland Veterans Affairs Research Foundation, Portland, OR USA; 2 The Hemophilia Center, Oregon Health & Science University, Portland, OR USA; 3 Medical Research Service, Portland Veterans Affairs Health Care System, 3710 SW US Veterans Hospital Road, Portland, OR 97239 USA; 4 Division of Endocrinology, Diabetes & Clinical Nutrition, Oregon Health & Science University, Portland, OR USA

**Keywords:** Hemophilia, Osteoporosis, Skeletal fragility, Thrombin, Coagulation, Clotting factor

## Abstract

Osteopenia and osteoporosis have increasingly become a recognized morbidity in those persons with hemophilia (PwH) receiving inadequate prophylactic clotting factor replacement. Animal models can control or eliminate genetic and environmental factors and allow for invasive testing not clinically permissible. Here, we describe the skeletal phenotype of juvenile and adult male mice with a genetically engineered deficiency in coagulation factor IX (FIX KO). Although the somatic growth of FIX KO mice matched that of their wild-type (WT) littermates at 10 and 20 weeks of age, the FIX KO mice displayed reduced bone mineral density (BMD), reduced cortical and cancellous bone mass, and diminished whole bone fracture resistance. These findings coupled with parallel observations in a murine model of hemophilia A (FVIII deficiency) point to an effector downstream of the coagulation cascade that is necessary for normal skeletal development. Further study of potential mechanisms underlying the bone disease observed in rare clotting factor deficiency syndromes may lead to new diagnostic and therapeutic insights for metabolic bone diseases in general.

## Introduction

Hemophilia is a rare congenital bleeding disorder characterized by prolonged and excessive bleeding after minor trauma or even occurs spontaneously at times [1]. The condition is inherited as a defect or mutation in the gene for a clotting factor. The most common forms of hemophilia are hemophilia A and hemophilia B that result from factor VIII (FVIII) and factor IX (FIX) protein deficiency or dysfunction, respectively [2, 3]. Hemophilia A is more prevalent (80%–85% of the total hemophilia population) than hemophilia B. It presents in 1 in 5000 live male births, whereas hemophilia B presents in 1 in 30,000 live male births [4]. The genes-encoding FVIII and FIX reside in the long arm of chromosome X and so both hemophilia A and B are inherited via an X-linked recessive pattern. Consequently, 100% of females born from affected fathers will be carriers, and none of the males born will be affected. Female carrier mothers have a 50% chance of having affected males and a 50% chance of having carrier females.

FVIII and FIX play essential roles in the process of blood coagulation. These clotting factors circulate as inactive enzyme precursors, but, upon activation, they form a tenase complex capable of activating factor X (FX) in the presence of Ca^2+^. Activated FX converts prothrombin to thrombin, a pivotal step necessary for clot formation. When either FVIII or FIX is deficient or dysfunctional, the coagulation cascade cannot activate appropriately, thus, abrogating the process of clot formation [1].

Prior to the development of factor concentrates in the mid-to-late 20th Century, the life expectancy of people who had severe hemophilia was only 11 years [3, 5]. Most people with severe hemophilia died in early childhood or adolescence from hemorrhage within vital organs. Now with the widespread availability of replacement therapy to prevent and treat active bleeding, improved management of blood-borne infections through surveillance, and effective treatment options for hepatitis C and HIV, affected individuals can expect a normal life span [6]. The change in the age distribution for persons with hemophilia (PwH) has led to the recognition of other comorbidities related to hemophilia [7, 8]. A large and growing body of literature has revealed an increased prevalence of skeletal fragility in both children and adults with hemophilia. Initially, the reduced bone mineral density (BMD) in PwH was attributed to decreased physical activity, recurrent hemarthroses resulting in debilitating arthropathy, and infection with either human immunodeficiency virus or hepatitis C [9]. More recently, basic and clinical work suggests that the reduced bone mass in PwH could be directly linked to FVIII and/or FIX deficiency. There is mounting evidence that thrombin is a crucial participant in processes outside of the hemostatic system [10]. Osteoblasts, the cells responsible for bone formation, express thrombin receptors, and thrombin has been shown to stimulate differentiation and activity of these cells [10, 11]. Consequently, a deficiency of either FVIII or FIX could exert unappreciated effects, including decreased BMD that are not directly related to bleeding or factor transfusion-related complications.

To explore the possibility of a direct effect of clotting factors on skeletal physiology, we have taken advantage of murine models of hemophilia [12, 13]. Despite exhibiting negligible factor levels, genetically engineered knockout (KO) FVIII and FIX mice do not bleed spontaneously [14]. Hence, these models allow one to examine the direct effect of FVIII or FIX on bone health, independent of joint bleeds, and other confounding variables. We have previously shown that FVIII knockout mice exhibit decreased bone mass and strength compared to intact littermates. To determine if the deleterious effect of FVIII deficiency is unique or persists in the setting of a different genetic form of hemophilia, we examined the skeletal consequences of FIX deficiency in one group of juvenile male mice (10-week-old) and another group of adult (20-week-old) male mice. With these studies, we hope to dissect the role of clotting factors in the abnormal skeletal phenotype observed in hemophilia. The findings show that male mice with a lifelong deficiency of FIX demonstrate impaired skeletal integrity comparable to that observed in FVIII-deficient male mice and the skeletal consequences are apparent long before adulthood.

## Materials and Methods

### Animals

FIX-deficient (FIX KO) breeder mice were generously provided by Professor Paul Monahan (University of North Carolina) and backcrossed with C57BL/6 J mice (B6). All male FIX KO mice and their male wild-type (WT) littermates were bred under identical conditions at the Portland Veterans Affairs Veterinary Medical Unit. At the time of weaning, male mice were group housed (2–4 animals per cage) and maintained with ad libitum water and laboratory rodent chow (Purina Diet 5001; PMI Feeds, Inc) in a 12-h light and 12-h dark cycle (6 am–6 pm). Bedding was changed on a weekly basis. Animals were handled minimally and by the tail to prevent bleeding and injury.

### Tissue Collection

FIX KO mice and their WT littermates were assessed and euthanized at 10 weeks of age and another group at 20 weeks of age (peak bone mass). Body lengths (defined as the distance from the tip of the nose to the base of the tail) were measured and weights were recorded. Blood samples were collected for biochemical analyses by cardiac puncture from mice under isoflurane anesthesia before cervical dislocation. The femora and tibiae were harvested immediately from each mouse. The right femur was measured from the greater trochanter to the external condyle using a digital caliper. After ex vivo dissection of soft tissue and fat, the excised femora were wrapped in sterile gauze soaked in phosphate-buffered saline and stored at − 20 °C until subsequent analyses. Tibiae were stored in 70% ethanol at 4 °C in sealed glass vials until analyses.

### PCR Genotyping

Conventional PCR assay methods were used to identify FIX KO genotype from WT littermates by collecting ear punches at weaning. No adverse bleeding events were observed from this tissue collection in FIX KO mice. DNA was isolated using REDExtract-N-Amp Tissue PCR Kit. (Sigma Aldrich, St. Louis, MO, USA). Standard oligonucleotide primer sets were used to identify the KO genotype Forward, 5′-GAGAACCTGCGTGCAATCCATCTTG-3′ and reverse, 5′-CCATTTCCACCTTCTCTTCCCACACG-3′ along with negative control primer set 5′-CACCTTGGAACGATCCTGTACTGAGC-3′, and reverse, 5′-GGAGTCACCTCTCTAGTTCCACACTC-3′ to identify the WT genotype [15]. 13uL of sample was run out on a 2% agarose gel. FIX KO (homozygous) mice were identified by the expected amplification product (about 512 bp) compared to WT (about 608 bp).

### Body Composition and BMD

Body composition (lean and fat mass) and areal bone mineral density (aBMD) measurements (mg/cm^2^) of the whole body and femora were determined by dual-energy X-ray absorptiometry using the PIXImus instrument (Lunar Corp). Densitometric analysis of the whole body (defined as the whole-body image minus the calvarium, mandible, and teeth) was performed on animals under isoflurane anesthesia. Routine calibration was performed daily with a defined standard (phantom). Previous experience with this instrument indicates a precision error (expressed as the coefficient of variation) for bone mineral content of 0.99 ± 0.51% and for BMD of 1.71 ± 0.33%.

### Cortical and Cancellous Bone Geometry and Microarchitecture

Three-dimensional microarchitectural changes in cortical and cancellous bone were assessed using quantitative X-ray microcomputed tomography (μCT). Cortical bone was evaluated in the mid-shaft of the femur, and cancellous bone was evaluated in the proximal tibia metaphysis. Cortical femoral shaft bone geometry was examined with a desktop X-ray microtomographic scanner (SkyScan Model 1074, Aartselaar, Belgium). Images were analyzed with Optimas software (version 6.2; Media Cybernetics, Silver Spring, MD). The cortical femoral shaft measurements included total cross‐sectional area, calculated as area of bone plus marrow enclosed within the periosteal surface; marrow area, defined by the endosteal surface; and cortical bone area, calculated as the difference between cross‐sectional area and marrow area. Cortical thickness was measured at eight sites equally spaced around the centrum, and an average thickness was calculated. Assuming rectangular pixels of height *h* and width *w*, with the centroid of the pixel located at a distance *d* from the neutral axis of the cross section, areal moment of inertia in the plane of bending (*I*_xx_) was calculated according to Turner and Burr [16]. Tibiae were scanned in 70% ethanol using a Scanco μCT40 scanner (Scanco Medical AG) at a voxel size of 12 × 12 × 12 μm (55-kVp X-ray voltage, 145-μA intensity, and 200-ms integration time). Filtering parameters, sigma and support, were set to 0.8 and 1, respectively. Forty slices (480 μm) of trabecular (cancellous) bone at the tibial metaphysis were evaluated for measurement of bone volume/tissue volume (BV/TV, %), trabecular number (Tb.N, m/m^1^), trabecular thickness (Tb.Th, μm), trabecular separation (μm), and connectivity density (Conn.D).

### Biomechanical Strength

To assess femoral structural properties, mouse femora were tested to failure in 3-point bending with a high-resolution materials test apparatus (Model 4442; Instron Corp). The loading fixture consisted of 2 fixed lower supports, placed at a span length of approximately 7 mm, and an upper loading point attached to a moving actuator. The upper loading point contacted the specimen at its midpoint, which was coincident with the center of the span. System software was used to displace the actuator at a strain rate of 0.5%/s until failure occurred. Load and displacement data were recorded, and failure load, representing the energy absorbed before breaking, and stiffness, calculated from the linear portion of the load vs displacement curve, were determined using system software. Strength and modulus were calculated using the cross‐sectional areal measurements previously determined by μCT.

### Measurement of Biochemical Parameters

SigmaFast Phosphatase Substrate Kit (Sigma Aldrich, St. Louis, MO, USA) containing PNPP (*p*-nitrophenyl phosphate disodium salt) was used to quantify serum alkaline phosphatase activity. Serum osteocalcin was measured using a commercial mouse osteocalcin ELISA kit (Quidel Corporation, CA, USA). All samples and standards were measured in duplicate.

### Statistical Analysis

All data are presented as the mean ± SEM, and statistical comparisons of experimental groups were evaluated by unpaired Student’s *t* test. A value of *p* < 0.05 was considered statistically significant.

## Results

**Fig. 1:**
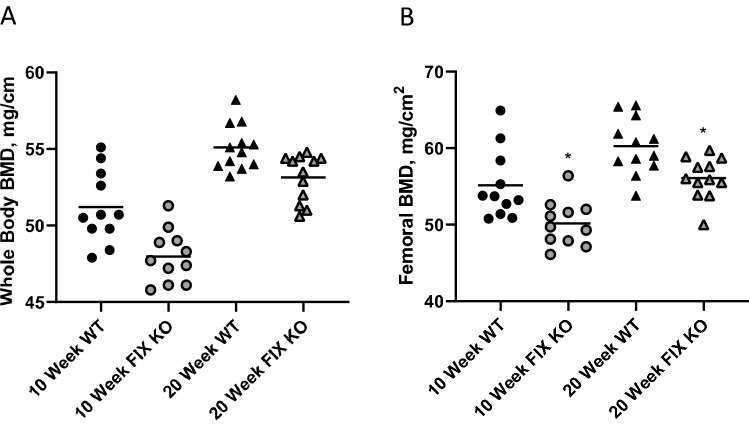
Juvenile (10-week-old) and adult (20-week-old) factor IX knockout (FIX KO) male mice exhibit reduced areal bone mineral density (BMD) compared to their wild-type (WT) littermates (panel **A** whole body; panel **B** whole femur). Individual data are presented to demonstrate the phenotype distribution. WT mice represented as black symbols and FIX KO mice were represented by gray symbols. Mean values are indicated by horizontal lines within each group. Statistical significance determined by Student’s *t* test (* indicates *p* value < 0.05)

General somatic features of juvenile and adult FIX KO mice were indistinguishable from their littermates in terms of body weight, lean and fat mass, and body length (Table 1). Hemarthroses events are rare in mice, because they do not have spontaneous bleeds. Animals included in this study showed no evidence of injury by change in behavior or activity levels during animal wellness checks; however, FIX KO mice show marked reductions in whole body and whole femoral BMD at both 10 weeks and 20 weeks of age (Fig. 1). In 10-week-old mice, whole-body BMD was 51.0 ± 0.8 mg/cm^2^ in WT and 48.0 ± 0.5 mg/cm^2^ in FIX KO (*p* < 0.005), and femoral BMD was 54.7 ± 1.5 mg/cm^2^ in WT and 50.2 ± 0.9 mg/cm^2^ in FIX KO (*p* < 0.05). In 20-week-old mice, whole-body BMD was 54.8 ± 0.5 mg/cm^2^ in WT and 53.1 ± 0.4 mg/cm^2^ in FIX KO (*p* < 0.05) and femoral BMD was 59.2 ± 1.4 mg/cm^2^ in WT and 55.6 ± 0.7 mg/cm^2^ in FIX KO (*p* < 0.05).

**Table 1:** Selected skeletal parameters in juvenile (10-week-old) and adult (20-week-old) factor IX knockout (FIX KO) mice compared to their wild-type (WT) littermates

	10-Week-old male mice			20-Weeek-old male mice		
Genotype	WT	FIX	*p value*	WT	FIX KO	*p* value
No. mice	11	11		12	12	
Somatic measures						
Body weight (g)	25.2 ± 0.6	23.9 ± 0.5	*n.s*	28.5 ± 0.6	28.1 ± 0.3	*n.s*
Lean mass (g)	21.9 ± 0.5	20.6 ± 0.4	*n.s*	24.3 = 0.5	23.7 ± 0.2	*n.s*
Fat mass (g)	3.3 ± 0.1	3.3 ± 0.1	*n.s*	4.2 ± 0.2	4.3 ± 0.1	*n.s*
Body length (cm)	9.6 ± 0.1	9.4 ± 0.1	*n.s*	9.9 ± 0.1	9.8 ± 0.1	*n.s*
Femoral length (mm)	15.4 ± 0.1	15.1 ± 0.1	< 0.05	15.8 ± 0.1	15.8 ± 0.1	*n.s*
Bone mineral density (BMD) measures						
Whole body BMD (mg/cm^2^)	51.0 ± 0.8	48.0 ± 0.5	< 0.005	54.8 ± 0.5	53.1 ± 0.4	< 0.05
Femoral BMD (mg/cm^2^)	54.7 ± 1.5	50.2 ± 0.9	< 0.05	59.2 ± 1.4	55.6 ± 0.7	< 0.05
Bone microanatomical measures						
Femoral mid-shaft diaphysis						
Cortical area (mm^2^)	0.673 ± 0.023	0.617 ± 0.016	< 0.05	0.771 ± 0.016	0.730 ± 0.013	< 0.05
Marrow area (mm^2^)	1.227 ± 0.044	1.010 ± 0.028	< 0.001	1.226 ± 0.033	1.130 ± 0.035	< 0.05
Total cross-sectional area, (mm^2^)	1.900 ± 0.064	1.627 ± 0.032	< 0.005	1.997 ± 0.046	1.852 ± 0.040	< 0.05
Moment of inertia (mm^4^)	0.120 ± 0.009	0.092 ± 0.004	< 0.01	0.135 ± 0.005	0.118 ± 0.006	< 0.05
Cortical thickness (μm)	146 ± 3	146 ± 4	*n.s*	164 ± 2	161 ± 2	*n.s*
Tibial proximal metaphysis						
Bone volume/tissue volume (%)	18.75 ± 1.19	16.91 ± 1.19	*n.s*	19.08 ± 1.04	15.96 ± 0.92	< 0.05
Connectivity density (1/mm^3^)	233.5 ± 16.3	220.8 ± 16.3	*n.s*	140.6 ± 6.8	109.9 ± 7.0	< 0.01
Trabecular number (1/mm)	6.93 ± 0.14	6.82 ± 0.14	*n.s*	5.71 ± 0.09	5.34 ± 0.12	< 0.05
Trabecular spacing (μm)	136.5 ± 3.4	141.0 ± 3.4	*n.s*	166.4 ± 3.0	180.6 ± 4.6	< 0.05
Trabecular thickness (μm)	44.0 ± 1.2	42.2 ± 1.2	*n.s*	48.6 ± 0.8	48.5 ± 1.0	*n.s*
Whole femoral biomechanical measures						
Ultimate failure load (N)	19.1 ± 1.1	15.9 ± 0.7	< 0.05	22.4 ± 0.4	20.0 ± 0.4	< 0.001
Stiffness (N/mm)	89.4 ± 4.5	74.1 ± 4.6	< 0.05	125.0 ± 5.8	100.6 ± 3.4	< 0.005
Strength (MPa)	167.6 ± 3.6	164.4 ± 5.1	*n.s*	173.1 ± 4.7	171.6 ± 5.6	*n.s*
Modulus (MPa)	4665 ± 162	4992 ± 276	*n.s*	5754 ± 220	5353 ± 272	*n.s*
Serum biochemistry measures						
Osteocalcin (ng/ml)	71.6 ± 5.5	63.3 ± 3.6	*n.s*	31.7 ± 3.1	36.8 ± 3.5	*n.s*
Alkaline phosphatase activity (μmol PNP/min)	42.0 ± 1.8	43.6 ± 1.8	*n.s*	30.4 ± 1.3	30.6 ± 1.4	*n.s*

Data are presented as mean ± standard error of the mean. Statistical significance determined by Student’s *t* test

**Fig. 2:**
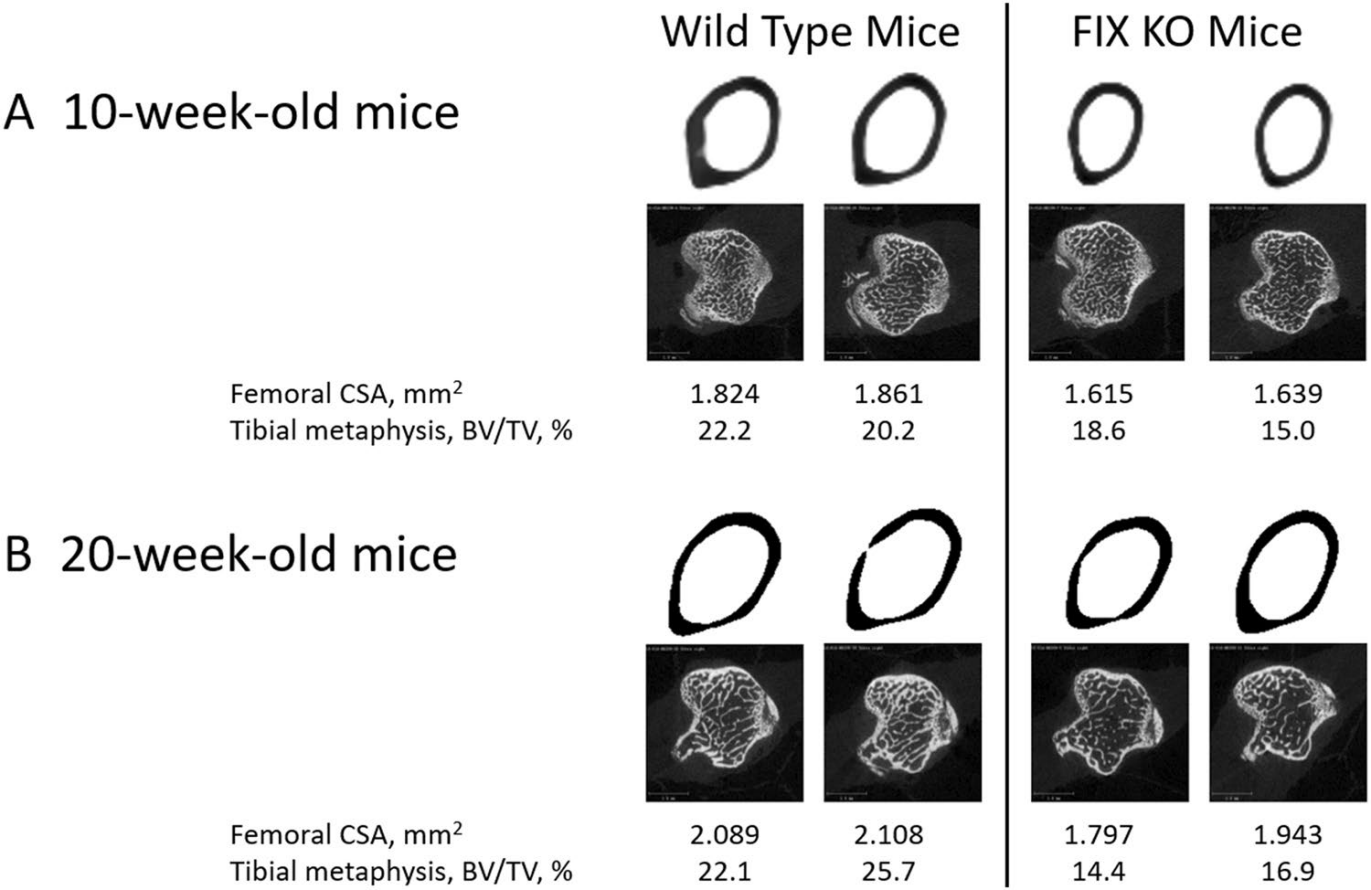
Juvenile (10-week-old) and adult (20-week-old) factor IX knockout (FIX KO) male mice exhibit reduced femoral cross-sectional area (CSA) and tibial metaphyseal bone volume relative to total volume (BV/TV). (panel **A** 10-week-old mice; panel **B** 20-week-old mice). Representative slices of femoral mid-shaft diaphysis (upper images of each panel) and tibial metaphysis (lower images of each panel) from two mice of each group are presented along with the measured values assigned to each scan

To investigate the structural differences underlying the observed reductions in BMD that accompany FIX deficiency, we analyzed representative compartments of cortical (femoral mid-shaft) and trabecular (tibial metaphysis) bone with microcomputed tomography (μCT) (Table 1 with representative examples presented in Fig. 2). At 10 weeks, the mean cortical thickness at the femoral mid-shaft was identical between FIX KO mice and their littermates (Table 1). However, all other areal measures were substantially reduced in the FIX KO mice with 14% reduced total cross-sectional area (*p* = 0.001) that was a consequence of an 8.4% difference in cortical area (*p* = 0.05) and 17% difference in marrow area (*p* < 0.001) (Table 1). The calculated femoral moment of inertia (*I*_xx_, a measure of resistance to bending failure) was also reduced 23.4% in 10-week-old FIX KO mice (*p* < 0.01) (Table 1). We next examined older 20-week-old mice. Consistent with the expected skeletal changes that accompany somatic growth and achievement of peak bone mass, cortical bone areas were greater in the 20-week-old mice compared to the 10-week-old mice in both the FIX KO and WT mice. And, just as was observed in the juvenile mice, total cross-sectional area, cortical area, marrow area, and moment of inertia were all found to be reduced in the adult 20-week-old FIX KO mice compared to their littermates, while mean cortical thickness was again found not to differ between FIX KO and WT mice. Two-way analysis of variance indicated statistically significant effects of both age and genotype on measures of cortical geometry but no interaction between age and genotype (data not shown).

Assessment of differences at the tibial metaphysis also demonstrated substantial reductions in the microarchitectural parameters of cancellous (trabecular) bone in FIX KO mice compared to their littermates. Bone volume (BV/TV; % of tissue area), trabecular number, connectivity density, and trabecular separation were all reduced in FIX KO mice, while there was no impact of genotype on trabecular thickness (Table 1). The differences in cancellous bone structure between FIX KO and WT mice were analogous at both time points, but statistically different measures were only confirmed in 20-week-old mice (Table 1).

**Fig. 3:**
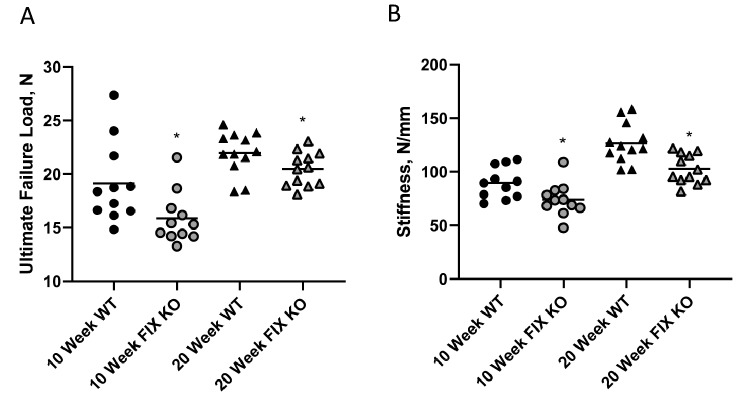
Juvenile (10-week-old) and adult (20-week-old) factor IX knockout (FIX KO) male mice exhibit reduced biomechanical measures of femoral bone strength determined by resistance to 3-point bending compared to their wild-type (WT) littermates (panel **A** ultimate failure load; panel **B** stiffness). Individual data are presented to demonstrate the phenotype distribution. WT mice represented as black symbols and FIX KO mice were represented by gray symbols. Mean values are indicated by horizontal lines within each group. Statistical significance determined by Student’s *t* test (* indicates *p* value < 0.05)

BMD is a strong predictor of fracture resistance. To test for effects of FIX deficiency on bone strength and stiffness, excised femora from 10- and 20-week-old FIX mice and their littermates were subjected to three-point bending. Consistent with the group differences found in BMD, ultimate failure load and stiffness at the femoral shaft were reduced in the FIX KO mice (at both 10 and 20 weeks) compared to their age-matched littermates (Fig. 3). At 10 weeks of age, ultimate failure load was 19.1 ± 1.1 N in WT and 15.9 ± 0.7 N in FIX KO (*p* < 0.05), and femoral stiffness was 89.4 ± 4.5 N/mm in WT and 74.1 ± 4.6 N/mm in FIX KO (*p* < 0.05). At 20 weeks of age, the differences in biomechanical measures of strength persisted with ultimate failure load 22.4 ± 0.4 N in WT and 20.0 ± 0.4 N in FIX KO (*p* < 0.0005) and femoral stiffness was 125.0 ± 5.8 N/mm in WT and 100.6 ± 3.4 N/mm in FIX KO (*p* < 0.005). The reduced failure load and stiffness in FIX mice are consistent with their smaller moment of inertia. No genotype-dependent differences in either tissue strength or modulus were observed. We interpret these findings to suggest that the reduced femoral failure load and stiffness as well as the reduced areal BMD measures observed in the FIX KO mice were a consequence of reduced bone size rather than any demonstrable changes in intrinsic material properties of the bone tissue.

Bone homeostasis and skeletal integrity are dependent upon the coordinated activities of osteoclasts, osteoblasts, and osteocytes. No genotype-dependent differences were present in markers of osteoblast function—circulating alkaline phosphatase activity and osteocalcin (Table 1).

## Discussion

Understanding the mechanism of decreased BMD and increased skeletal fragility in the hemophilia population is critically important, especially now that those with hemophilia can have a normal life expectancy. Previous in vivo investigations of a murine model of hemophilia A showed that complete factor VIII deficiency is associated with a congenital osteoporotic phenotype in the absence of injury or observed hemorrhage [17–19]. As just one facet of the coagulation cascade was examined in those experiments, no inference could be made as to whether the observed abnormal bone homeostasis was mechanistically attributable to a specific property of the FVIII molecule versus defective hemostasis, inflammation, or some other more global phenomenon [20, 21].

Utilizing a murine knockout disease model of hemophilia B (Christmas disease), we have demonstrated that FIX is also critically important to normal bone development. Compared to their WT littermates, mice lacking FIX exhibit lower BMD, reduced measures of cortical and trabecular bone mass, and femora less resistant to fracture. Our findings are similar to, and extend, those of Taves et al. [22] who previously explored the skeletal response to experimentally induced joint injury in adult (22-week-old) FIX KO mice. Although observations in mouse models cannot directly translate to human clinical data, animal studies do provide the experimental control of genetic and physiological manipulations essential to explore mechanisms that contribute to disease vulnerability. The exact mechanism(s) underlying reduced BMD in PwH is certainly not yet understood, but together these experimental findings identify a normally functioning coagulation cascade as an essential physiological requirement for skeletogenesis.

Components of the coagulation cascade originally studied for their role in hemostasis are now recognized as key players in various pathophysiologic and biological processes [23–26]. Absent levels or defective function of either FVIII or FIX impairs FX activation resulting in deficient thrombin generation. Both osteoblasts and osteoclasts express the thrombin receptor (F2R or PAR1) [10, 27–29], and in vitro studies indicate that thrombin induces differentiation of osteoblasts [29–32] and negatively regulates osteoclast formation and function [33, 34]. In support of this mechanistic hypothesis, Aronovitch et al. [35] demonstrated complete knockout of either FVIII or PAR1 in mice resulted in similar bone structural abnormalities.

Another recognized outcome of defective thrombin generation is decreased activation of FXIII [36]. Once activated FXIII functions as a transglutaminase [25, 37]. Transglutaminases are a family of enzymes that catalyze γ-glutamyl-ɛ-lysyl crosslinks within their specific substrate proteins. Crosslinking in this way can stabilize molecular structures, increase protein matrix formation, and enhance cell adhesion. Crosslinks of the structure γ-glutamyl-ε-lysine are abundant in bone matrix. Fibronectin, a ubiquitous extracellular matrix protein required for hard tissue development, matrix maturation, and mineralization, is a well-known substrate for TGs, particularly TG2 and FXIII. Mice lacking both TG2 and FXIII-A exhibit increased bone marrow adipogenesis and osteoclastogenesis in vivo and reduced bone mass and biomechanical properties in both cortical and cancellous bone [38]. However, mice lacking FXIII alone display a normal skeletal phenotype suggesting that the expression of TG2 (or other TGs) may be increased in compensation. In humans, FXIII deficiency is a rare-inherited bleeding disorder with a frequency in the general population approximately 1:2,000,000 [39], and there are no clinical reports examining the skeletal status of patients with this particular bleeding disorder. It is evident that further work is necessary to define the role of FXIII in the formation, maintenance, and repair of mineralized tissues in vivo.

This report is the first to examine the impact of a congenital coagulation factor deficiency on the skeletal changes that accompany the transformation from growing (juvenile) to skeletally mature (adult) status. In mice, as in humans, bone size, mass, and strength increase during the transition from adolescence to adulthood. In our study, we found no evidence that FIX deficiency predisposed to inhibition of either somatic growth or bone elongation between the ages of 10 and 20 weeks. Furthermore, all of the features of impaired skeletal integrity observed in the adult 20-week-old mice (reduced mass, density and biomechanical strength) were already manifest in the 10-week-old mice. Our group has previously reported that the decreased BMD seen in the FVIII KO mice is likely due to increased bone resorption based on histomorphometric analysis that demonstrated no difference in most indices of bone formation and mineralization (*i.e.,* osteoblast‐lined bone perimeter, mineralizing perimeter, mineralization rate, and bone formation rate adjusted to bone area) but a significant increase in the osteoclast‐lined bone perimeter in the FVIII KO mice [18]. Unfortunately, we were unable to employ bone histomorphometric analysis in this experiment. A similar imbalance in bone cell function affecting skeletal geometry may be responsible for the osteoporotic phenotype present in FIX deficiency as well. However, further investigation with studies at varying time points is needed to determine if the decreased bone health observed in FIX-deficient mice is due to a biological impairment at a specific developmental stage that persists through adulthood or if there are other imbalances in bone homeostasis resulting from the specific absence of FIX.

The present study is not without its limitations. At the earliest time point, we examined (10 weeks) that skeletal pathology was already evident in the FIX KO mice. An evaluation of earlier time points (*e.g.,* immediately post-partum and at conclusion of weaning) as well as in aged mice (e.g., 52 weeks or older) would have provided additional information concerning the impact of a coagulation defect on the skeleton during the physiologic changes that accompany intrauterine growth, lactation, and aging. The RANKL/OPG system is a major signaling pathway regulating the differentiation and function of osteoblasts and osteoclasts [40–42]. Unfortunately, technical difficulties precluded the reliable assessment of these molecules.

In conclusion, by taking advantage of experimental animal models, we [18, 19] and others [17, 22, 43] have established that a deficiency in either factor IX or factor VIII in mice reproduces the decreased BMD and increased skeletal fragility that are observed in PwH. These mouse models allowed for the elimination of clinically confounding effects (*i.e.,* reduced physical activity, hemarthroses, other comorbid conditions), thereby lending strong support to the proposition that a fully functioning coagulation cascade is a physiological necessity to insure normal skeletal development. Further studies are needed to explore the underlying mechanism by which a defective coagulation cascade and the resulting thrombin deficiency alters normal skeletogenesis. Such work may identify a previously unappreciated role of the blood clotting system in regulating skeletal health and lead to the development of novel treatment strategies for osteoporosis in settings beyond those of hemophilia.
